# Piezoelectric-hydrogen synergy in electrospun oriented fibrous scaffold enables multimodal repair of long-distance peripheral nerve defect

**DOI:** 10.1093/rb/rbag156

**Published:** 2026-07-21

**Authors:** Haonan Gu, Shuang Zhao, Yating Gou, Yutong Yuan, Yue Li, Haoran Ren, Yan Jin, Biyun Li, Hongyun Xuan, Ye Xue, Peipei Liu, Zao Dai, Huihua Yuan

**Affiliations:** School of Life Sciences, School of Information Science and Technology (Tongke School of Microelectronics), Nantong University, Nantong 226019, China; School of Life Sciences, School of Information Science and Technology (Tongke School of Microelectronics), Nantong University, Nantong 226019, China; School of Life Sciences, School of Information Science and Technology (Tongke School of Microelectronics), Nantong University, Nantong 226019, China; School of Life Sciences, School of Information Science and Technology (Tongke School of Microelectronics), Nantong University, Nantong 226019, China; School of Life Sciences, School of Information Science and Technology (Tongke School of Microelectronics), Nantong University, Nantong 226019, China; School of Life Sciences, School of Information Science and Technology (Tongke School of Microelectronics), Nantong University, Nantong 226019, China; School of Life Sciences, School of Information Science and Technology (Tongke School of Microelectronics), Nantong University, Nantong 226019, China; School of Life Sciences, School of Information Science and Technology (Tongke School of Microelectronics), Nantong University, Nantong 226019, China; School of Life Sciences, School of Information Science and Technology (Tongke School of Microelectronics), Nantong University, Nantong 226019, China; School of Life Sciences, School of Information Science and Technology (Tongke School of Microelectronics), Nantong University, Nantong 226019, China; Department of Neurology, Clinical Medical College, Yangzhou University, Yangzhou 225001, China; School of Life Sciences, School of Information Science and Technology (Tongke School of Microelectronics), Nantong University, Nantong 226019, China; School of Life Sciences, School of Information Science and Technology (Tongke School of Microelectronics), Nantong University, Nantong 226019, China

**Keywords:** hydrogen, topographical guidance, piezoelectric stimulation, regenerative microenvironment, peripheral nerve regeneration

## Abstract

Functional recovery after long-distance peripheral nerve injury is limited by a poor regenerative microenvironment and slow axonal growth. This study introduces a multifunctional neural scaffold combining topographical, piezoelectric and hydrogen-release strategies. Fabricated via stable jet electrospinning from poly-L-lactic acid, poly(ethylene oxide) and siliconized magnesium (PLLA/PEO/Mg_2_Si), the oriented fiber scaffold mimics aligned microstructures, provides controllable piezoelectric signals and enables sustained hydrogen delivery. *In vitro*, it scavenged reactive oxygen species, supported aligned Schwann cell extension and promoted pro-regenerative M2 macrophage polarization. In a rat model with 15 mm sciatic nerve defects, early implantation reduced inflammation, accelerated axonal regeneration and myelination, improved electrophysiological recovery and reduced muscle atrophy. Mechanistically, piezoelectric modulation of the local electrical microenvironment contributed to these effects. By integrating electro-morpho-chemical signaling, this ‘piezoelectric-orientation-hydrogen’ scaffold offers a promising strategy for repairing long-segment peripheral nerve injuries.

## Graphical abstract

**Graphical Abstract:**
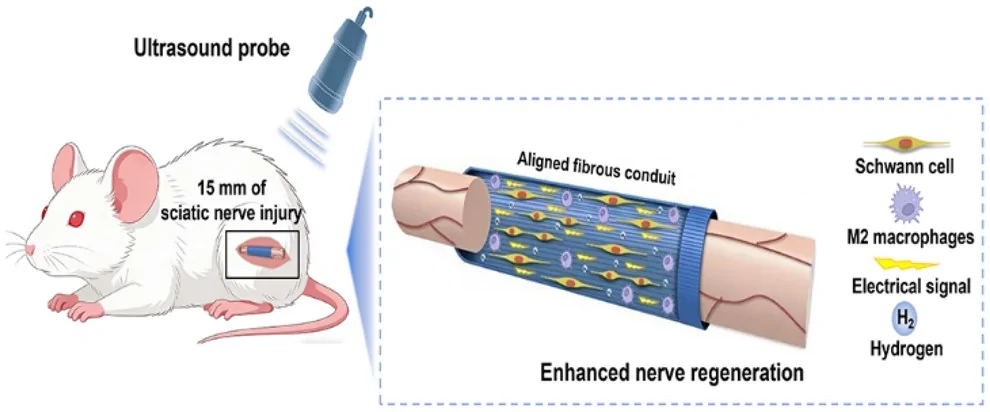

## Introduction

Peripheral nerve injuries, frequently resulting from trauma, tumor resection or iatrogenic causes, often lead to sensory and motor deficits and, in severe cases, permanent disability [1, 2]. The repair of extensive nerve defects continues to pose a substantial clinical challenge. While short-segment defects (<8 mm) can typically be addressed through direct nerve anastomosis, autologous nerve grafting remains the gold standard for longer defects. Nevertheless, this approach is constrained by limited donor availability, additional surgical trauma and potential donor-site morbidity, thereby limiting its widespread applicability. In this context, tissue engineering—leveraging functionalized biomaterial scaffolds and modulation of the regenerative microenvironment—has emerged as a promising alternative for large-segment nerve injuries unsuitable for direct suturing [3–5]. Consequently, the development of biomimetic tissue-engineered nerve grafts that integrate key features of the regenerative process (e.g. electrical signaling and inflammatory modulation) holds substantial theoretical and clinical value in promoting rapid axonal regeneration and functional recovery following long-distance peripheral nerve injury.

Accumulating evidence supports the potential of neural scaffolds in peripheral nerve regeneration. These constructs effectively bridge nerve gaps, guide directional axonal extension [6] and promote angiogenesis, myelination and synaptic reconstruction by modulating cellular behavior and providing paracrine support [7]. Despite these advances, current scaffolds often fail to replicate the anisotropic architecture of native neural tissue, a limitation that critically undermines their regenerative efficacy [8]. In response, researchers have developed various strategies to introduce anisotropic topographies onto fiber-based scaffolds. For instance, Fan and coworkers [9] successfully repaired a 15 mm sciatic nerve defect in rats using polycaprolactone (PCL) nerve conduits with oriented microstructures. Such microtopological cues mimic the biophysical features of the extracellular matrix (ECM), thereby guiding cell alignment and functional polarization, and have become a central focus in neural tissue engineering. Although scaffolds with unidirectional guidance or nanoscale topologies have demonstrated efficacy in promoting directed neurite outgrowth [10], their performance in restoring motor function and achieving axonal maturation in long-gap repairs remains inferior to that of autografts. Therefore, innovative engineering strategies that integrate multiple therapeutic modalities are urgently needed.

Recent years have witnessed the application of diverse biophysical approaches for peripheral nerve repair [11–13]. Among these, electrical stimulation has attracted considerable interest due to its capacity to promote axonal growth and remyelination [14, 15]. However, conventional electrical stimulation systems often rely on external wiring or implantable power sources, which pose risks of infection, device nondegradability and restricted patient mobility—factors that adversely impact postoperative recovery and quality of life [16, 17]. To address these challenges, indirect, power-free electrical stimulation strategies have emerged as a compelling alternative. These approaches generate electrical signals through material–physical interactions, such as those observed in magnetoelectric composites, which produce induced electric fields under external magnetic fields. Nevertheless, such systems require complex energy conversion pathways and deliver only single-frequency stimulation [18]. By contrast, piezoelectric biomaterials enable the direct conversion of ultrasonic mechanical energy into localized electric fields, offering a more efficient and wireless modality for electrical stimulation. Moreover, when combined with microfabrication/nanofabrication techniques, piezoelectric scaffolds can incorporate defined topological features, enabling dual modulation of bioelectrical and biomechanical cues aligned with tissue repair demands [19–21]. Although earlier studies have explored piezoelectric materials for nerve repair, these typically relied on spontaneous signals generated by tissue movement or axonal traction and often employed disordered material structures, limiting their capacity to guide long-distance regeneration under static conditions [22, 23]. Recent work utilizing highly oriented PLLA/PEG blends with radiofrequency stimulation has shown potential in peripheral nerve repair; however, such investigations remain confined to 8 mm defect models [24].

Poly-L-lactic acid (PLLA) is a biocompatible and biodegradable polymer whose degradation product, lactic acid, poses no risk to human health. It has been widely employed in surgical sutures, drug delivery systems and tissue engineering scaffolds [25, 26]. Electrospinning represents a mature technique for fabricating fibrous scaffolds that recapitulate the fibrillar architecture of the ECM, featuring high porosity and excellent interconnectivity conducive to nutrient diffusion and cellular infiltration, thereby facilitating tissue repair [27]. Our prior work has established PLLA as a bio-based piezoelectric polymer with high flexibility and ease of processing into membrane formats [28]. Accordingly, electrospun aligned PLLA fibers represent a promising candidate for neural tissue engineering scaffolds.

Tissue injury is frequently accompanied by an inflammatory response, with excessive production of reactive oxygen species (ROS) implicated in the pathogenesis of conditions including bacterial infection, sepsis, osteoarthritis and asthma [29, 30]—peripheral nerve injury being no exception [31]. Although ROS play essential roles in immune defense, such as macrophage-mediated pathogen elimination [32], their overproduction exacerbates tissue damage. Hydrogen gas (H_2_) exhibits selective antioxidant and anti-inflammatory properties, efficiently neutralizing highly cytotoxic ROS such as hydroxyl radicals into water, thereby mitigating oxidative injury while preserving signaling-relevant ROS [33, 34]. Even at elevated concentrations, H_2_ maintains excellent biocompatibility [35]. Its therapeutic efficacy has been validated across multiple inflammatory disease models, whether delivered via inhalation, oral hydrogen-rich water or hydrogen-rich saline injection [36]. However, the low aqueous solubility of H_2_ restricts its bioavailability at target lesions [37]. Achieving localized, sustained hydrogen release at sites of inflammation is therefore critical to maximizing therapeutic benefit and is considered a clinically favorable strategy [38]. For instance, Yang *et al*. [39] developed magnesium-doped PLLA composite microspheres, capable of controlled drug release and inflammation mitigation. Animal studies demonstrated reduced macrophage infiltration and diminished inflammatory cytokine expression, confirming the feasibility of using degradable magnesium to modulate drug release and suppress PLLA implant-induced inflammation. Similarly, two-dimensional Mg_2_Si nanosheets have been shown to sustainably generate hydrogen, effectively alleviating local inflammation and promoting tissue repair in deep burn skin models [40]. These findings suggest that hydrogen delivery may ameliorate the inflammatory microenvironment following nerve injury and thereby facilitate neural repair.

To date, most studies have confined their evaluations of electrospun nanofiber-based neural scaffolds to preliminary *in vitro* or *in vivo* assessments of surface properties. Only a limited number of studies have systematically integrated magnetoelectric materials with gas therapy. However, magnetoelectric composites are constrained to single-frequency electrical stimulation and often necessitate supplementary components to induce targeted tissue repair, leading to increasingly complex systems [41]. In contrast, piezoelectric biomaterials enable more direct and efficient wireless electrical stimulation. By incorporating hydrogen-generating particles, such scaffolds can simultaneously confer biomechanical, bioelectrical and gas-therapeutic effects—multifaceted regulation well aligned with tissue repair requirements. Furthermore, few studies have explored the mechanistic underpinnings of piezoelectric scaffold-mediated sciatic nerve repair, despite the essential role such mechanisms play in advancing peripheral nerve injury therapies.

**Figure 1:**
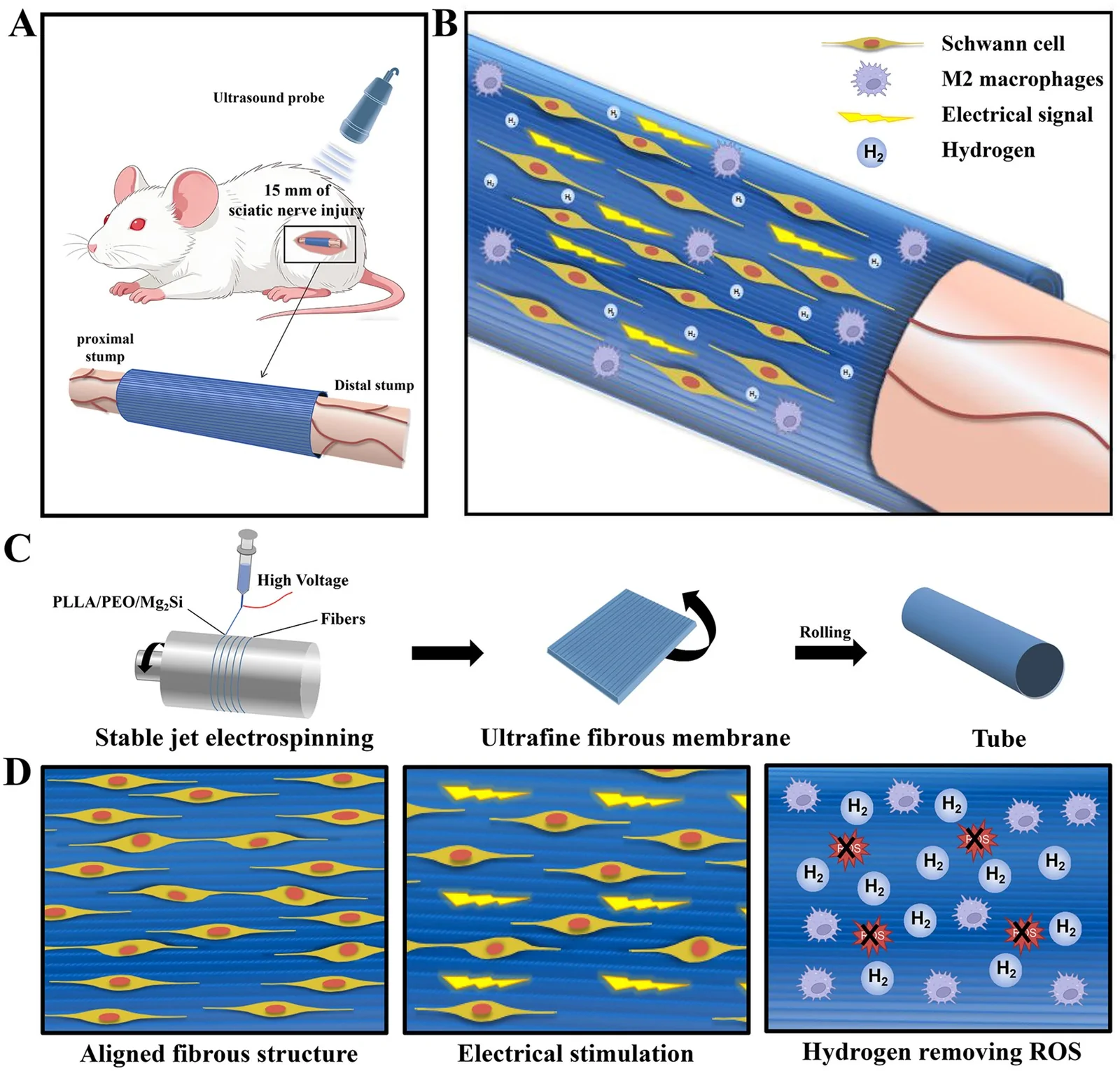
Schematic diagram illustrating the fabrication and application of PLLA/PEO/Mg_2_Si ultrafine fibers. (**A**) Schematic of ultrasound-driven PLLA/PEO/Mg_2_Si fiber scaffold for peripheral nerve injury regeneration. (**B**) Microenvironment of nerve injury within the PLLA/PEO/Mg_2_Si fiber scaffold. (**C**) Process flow for manufacturing PLLA/PEO/Mg_2_Si via electrospinning, followed by rolling the fabric into a tube. (**D**) Schematic illustrating the role of PLLA/PEO/Mg_2_Si in promoting nerve regeneration.

In this study, we aim to develop a neural scaffold with a simplified composition yet multifunctional capabilities. As shown in Figure 1, this scaffold embodies a combinatorial strategy that integrates the surface properties of aligned electrospun fibers with H_2_ therapy, synergistically activated by ultrasound-triggered bioelectric stimulation, for the repair of long-segment peripheral nerve defects. Using electrospinning technology, we successfully fabricated linearly aligned piezoelectric nanofiber membranes composed of PLLA, PEO and Mg_2_Si. PEO was incorporated to modulate the loading of Mg_2_Si [42], thereby conferring sustained hydrogen release and endowing the scaffold with a hydrophilic surface that enhances biocompatibility and piezoelectricity [24]. Importantly, the incorporation of Siliconized magnesium (Mg_2_Si) not only preserves fiber alignment but also significantly augments the scaffold’s piezoelectric properties and enables continuous hydrogen production. The resultant scaffold exhibits excellent biocompatibility, effectively inducing directed Schwann cell migration and promoting their neuroregenerative phenotype. Moreover, the hydrogen released from the scaffold efficiently scavenges ROS, contributing to the attenuation of nerve injury-induced inflammation via immunomodulatory effects, including the promotion of M2 macrophage polarization. Finally, we further elucidate the mechanism by which this piezoelectric scaffold facilitates sciatic nerve repair. Collectively, this work achieves synergistic neural tissue repair by integrating ultrasound-activated bioelectric stimulation with the anti-inflammatory effects of hydrogen, offering a promising strategy for peripheral nerve regeneration.

## Experimental section

### Materials

PLLA (*Mn* = 1 × 10^5^) was purchased from Jinan Daigang Biomaterials Co. Ltd. Poly(ethylene oxide) (*Mw* = 5 × 10^6^) was obtained from Alfa Aesar (China) Chemical Co., Ltd. Mg_2_Si was provided by Aladdin Reagents (Shanghai) Co., Ltd. Trifluoroethanol was purchased from Shanghai Darui Fine Chemicals Co.

### Preparation of oriented electrospun fibers

A solution comprising 0.425 g PLLA and 0.075 g poly(ethylene oxide) (*Mr* = 5000 kDa) was prepared by dissolving the components in 10 mL trifluoroethanol and stirring the solution at room temperature overnight. According to the ratio Mg_2_Si: PEO = 1:5, add 0.015 g of Mg_2_Si, and stir at room temperature overnight. The PLLA solution was employed to generate nanofibers with exceptional fineness under the influence of a high-voltage electric field within the context of high-voltage electrostatic spinning. The high-voltage power supply was maintained at 7–10 kV, and the solution push rate was 0.5 mL/h. The fiber filaments were collected using a high-speed rotating drum wrapped with aluminum foil paper at 2000 rpm, and the spinning process lasted for 6 h. The fiber film was removed and placed in a vacuum-drying oven for 48–72 h to remove excess organic reagents, resulting in oriented microfibers.

### Scanning electron microscope observation

The dried electrospun fibers were cut into small pieces and fixed on the sample stage using conductive adhesive tape to keep the sample surface flat. After spraying with gold, the fiber morphology was observed using an electric field emission scanning electron microscope (FE-SEM, ZEISS Gemini SEM 300) with a voltage of 5 kV and a grating of 30 nm.

### Mechanical properties

Mechanical properties were evaluated using a benchtop testing machine (ZQ-990LB; Zhichao Precision Instrument) equipped with a 500 N load cell. Cylindrical specimens (0.3–0.9 mm diameter) were stretched at a constant speed of 10 mm/min at room temperature. Tensile strength, Young’s modulus and fracture strain were derived from the stress–strain curves. Three parallel samples were tested per group.

### Contact angle

The hydrophilicity of the prepared nanofibers was tested using a JCY series contact angle analyzer (JCY-1, Shanghai Fangrui Instruments). A drop of water (0.25 mL) was added on the surface of the nanofiber membrane, and the droplet shape was recorded after 2 s. Each group was set up with three parallel samples.

### Piezoelectric property analysis

The fiber membrane was cut into 2 cm × 2 cm squares, with copper wires attached to both ends for signal conduction, and encapsulated with polyimide film. A pressure of ∼7 N was applied at a set frequency using a desktop testing machine equipped with a weighing sensor. The copper electrodes were connected to an electrochemical workstation to collect and record the generated electrical signals over time.

### Measurement of H_2_ release from PLLA/PEO/Mg_2_Si fiber membrane

Under the catalysis of platinum nanoparticles, methylene blue (MB) is rapidly reduced by H_2_ to colorless MBH_2_, allowing an MB‑Pt nanoparticle mixture to serve as a probe for H_2_ release. The fiber membrane is placed in a PBS solution together with the probe. The absorbance of MB in the release medium is measured at 665 nm by Ultraviolet–visible (UV-Vis) spectroscopy. Since the decrease in MB absorbance correlates linearly with the amount of H_2_, the H_2_ released from the PLLA/PEO/Mg_2_Si fiber membrane is calculated from the absorbance values using a standard MB calibration curve.

### POD-like activity of PLLA/PEO/Mg_2_Si fiber membranes

Peroxidase (POD) catalyzes the oxidation of H_2_O_2_ by ABTS, producing blue ABTS^+^. Enzyme activity is determined from absorbance changes. The ABTS substrate solution was prepared at 0.5 mmol/L in deionized water. The reaction mixture contained the fiber membrane, 30 μL ABTS solution, 20 μL 30% H_2_O_2_, and 400 μL HAc‑NaAc buffer. After preheating to 40°C, absorbance at 450 nm was recorded over 10 min using a multifunctional microplate reader.

### Cell experiments

Fibers treated for different durations were cut into 15 mm diameter circles and placed in 24-well cell culture plates, with tissue culture plates (TCP) as a blank control. After sterilization, RSC96 cells were seeded onto the material surfaces and cultured in DMEM high-glucose medium supplemented with 10% fetal bovine serum (FBS) and 1% penicillin–streptomycin (PS). Each group was subjected to ultrasonic stimulation using a cell-breaking apparatus (JY92-IIN) at 300 W for 5 min per day to enhance piezoelectric output.

### Biocompatibility testing of PLLA/PEO/Mg_2_Si fiber membranes

The plate was placed in a cell culture incubator for culture, with medium changes performed every other day. Add CCK-8 solution to the cell culture medium and incubate at 37°C for 2 h. After incubation, measure the absorbance at 450 nm using a microplate reader.

### Cellular immunofluorescence staining

After 3 days of culture, the medium was removed, and cells were rinsed three times with PBS. Cells were fixed in 4% paraformaldehyde for 10 min at room temperature, followed by three PBS washes. Permeabilization was performed with 0.1% Triton X-100 for 10 min, followed by three 5‑min PBS washes. Nonspecific binding was blocked with 1% bovine serum albumin (BSA) for 30 min. The S100 antibody was applied overnight at 4°C, followed by three 5‑min washes with PBST. The secondary antibody (diluted in 1% BSA) was incubated in the dark at room temperature for 1 h, then washed three times with PBS. A drop of DAPI-containing mounting medium was placed on a slide; the coverslip was removed from the well, inverted onto the slide and sealed with nail polish. For actin staining, 3‑day cultured Schwann cells were stained using Actin-Tracker Green-488.

### Quantitative reverse transcription-polymerase chain re-action(qPCR)

S100b mRNA expression was analyzed by qPCR. RSC96 cells (5 × 10^5^ cells/mL) were seeded into 6-well plates containing different materials and cultured for 3 days. Total RNA was extracted and purified, and qPCR was performed using a real-time PCR system. The thermal cycling protocol consisted of 50°C for 2 min and 95°C for 25 min, followed by 40 cycles at 60°C for 60 s. Relative transcript levels were calculated using the 2^-^^ΔΔCt^ method. S100 calcium-binding protein B (S100b): forward primer 5′-ACTGAGGGACGAAATCAACA-3′; reverse primer 5′-CAACG-GAGGTGCTATTGGTA-3′.

### PLLA/PEO/Mg_2_Si fiber membranes’ reactive oxygen species scavenging capacity in cells

ROS were detected using a commercial kit. DCFH-DA, a nonfluorescent lipophilic probe, freely crosses cell membranes and is hydrolyzed by intracellular esterases to DCFH, which accumulates inside cells. ROS oxidize DCFH to DCF, a green fluorescent compound. Fluorescence intensity correlates positively with ROS levels, allowing quantitative assessment. For staining, DCFH‑DA was diluted 1:1000 in serum‑free medium. Cells in 24‑well plates were incubated with 500 μL of this working solution at 37°C for 20 min, washed three times with serum‑free medium, and observed under a fluorescence microscope.

### Macrophage polarization

RAW264.7 cells were cultured in medium changed every 2 days. At approximately 80% confluence, cells were washed three times with PBS, detached by pipetting, centrifuged, resuspended in fresh medium, counted using a hemocytometer and seeded at 1 × 10^4^ cells/well in a 24‑well plate. After 1 day of culture, the medium was replaced with medium containing 1 μg/mL LPS to activate macrophages and induce inflammation. Following 24 h of stimulation, the LPS‑containing medium was removed, cells were washed three times with PBS, and fresh medium was added. After three more days, cells were washed three times with PBS and processed for immunofluorescence staining.

### Animal experiments

Male SD rats weighing approximately 200 g were obtained from the Animal Experimentation Center of Nantong University. All animal experiments conducted in this study were approved by the Nantong University Laboratory Animal Management Committee. The approval number was P20250305-009. Rats were anesthetized with anesthetics (300 mg/mL) at 1 mL per 100 g body weight. After anesthesia, the left thigh was shaved, and a superficial skin incision was made at the femur. The intermuscular fascia was identified as a distinct white line, a small incision was made along it, and the sciatic nerve was bluntly dissected using vascular forceps and blunt scissors. The nerve was then transected to create a 15 mm defect. In the positive control group, a 15 mm autograft was excised, rotated 180°, and sutured to the nerve stump. In the negative control group, a 15 mm silicone tube (without fibrous membrane) was directly sutured onto the damaged nerve. For two weeks postsurgery, rats received antibiotics in drinking water and were kept under a 12 h light–dark cycle. One week postsurgery, skin wound healing began. During weeks 2–3 after surgery, the nerve injury site received ultrasound therapy once daily at 5 W for 5 min per session.

### Paraffin sectioning and H&E staining of muscle tissue

After 12 weeks in rats with a nerve injury model, the gastrocnemius muscles from the operated (right) and nonoperated (left) sides were excised under anesthesia. The proximal end was severed at the femoral condyle attachments, the distal end at the Achilles tendon, and residual blood was cleared from the muscle surfaces. The gastrocnemius muscle wet weight ratio was calculated according to Equation (1):


(1) Gastrocnemius muscle wet weight ratio(%)=(Ms/Mc)×100%

where *Ms* was the wet weight of the ipsilateral gastrocnemius muscle, and Mc was the contralateral muscle.

For comprehensive analysis of gastrocnemius muscle recovery in 12-week-old rats, the muscle was paraffin-embedded, sectioned and stained with H&E and Masson’s trichrome to visualize muscle fibers and collagen fibers.

### Immunofluorescence staining of nerve tissue sections

At week 12, nerves were harvested from the experimental and control groups (PLLA/PEO, Mg_2_Si and Autograft). Rats were anesthetized intraperitoneally, and regenerated sciatic nerves were resected and fixed in 4% paraformaldehyde for 24 h. Tissues were then immersed in 20% sucrose solution for 24 h until they sank to the bottom, followed by 30% sucrose for 48 h. After dehydration, samples were blotted dry, briefly immersed in liquid nitrogen, and stored at −80°C. Nerves were subsequently cryosectioned and processed for immunofluorescence staining (S100 and NF200), followed by fluorescence intensity analysis.

### Sciatic nerve function recovery

Gait analysis was performed at 2, 4, 8 and 12 weeks postsurgery to assess sciatic nerve functional recovery. Hind paw pads were coated with ink, and rats were allowed to walk along a white paper-lined runway at a constant speed. The procedure was repeated three times per rat, and footprints were recorded. Nerve recovery was evaluated using the SFI, and the SFI was calculated according to Equation (2):


(2) $$\text{SFI}=\frac{-38.3\times (\text{EPL}-\text{NPL})}{\text{NPL}}+\frac{109.5\times (\text{ETS}-\text{NTS})}{\text{NTS}}+\frac{13.3\times (\text{EIT}-\text{NIT})}{\text{NIT}}-8.8,$$

where *N* was nonsurgical foot, and *E* was experimental foot, and pedicle length (PL) was the distance from the hindfoot to the top of the third toe, and intermediate toe extension (IT) was the distance between the 2nd and 4th toes, and toe spread (TS) was the distance between the 1st and 5th toes.

### Gait analysis of rats

At 12 weeks postsurgery, electrophysiological analysis was performed using a full‑function electromyography evoked potential recorder. Rats were anesthetized by intraperitoneal injection, and the hair on the leg was shaved to expose the injured sciatic nerve. A ground electrode was placed on the ear or tail, the stimulating electrode was positioned at the proximal end of the nerve injury site, and the recording electrode was inserted into the ipsilateral gastrocnemius muscle. Stimulation intensity was set to 5 mA, and each electrical stimulus induced gastrocnemius contraction. The resulting signal was recorded, baseline processed and the onset, end and peak amplitudes were marked before saving the data. The same procedure was repeated with the stimulating electrode placed at the distal end of the injury site.

### Levels of myelin regeneration in neonatal nerve tissue

Myelin regeneration was investigated using transmission electron microscopy. Following anesthesia, nerves were harvested from conduits or autologous nerves within minutes. Samples (approximately sesame seed‑sized) were precisely collected from the central segment of the regenerated nerve on ice. Tissues were fixed, washed, dehydrated and prepared for ultrathin sectioning. Newly formed myelin sheaths were examined under transmission electron microscopy, and sheath thickness was quantified.

### Transcriptomic studies on the effects of peripheral nerve regeneration

For RNA sequencing, sciatic nerve samples were collected 12 weeks post‑treatment, and the proximal nerve stump was cryopreserved in liquid nitrogen. Total RNA was extracted using TRIzol reagent (Thermo Fisher Scientific, USA) following the manufacturer’s protocol. Sequencing was performed by Azenta. Raw data were filtered with fastp, and cleaned data underwent quality control using FastQC; only data meeting quality standards were analyzed further. For biological replicates, differential expression analysis was conducted using DESeq2, with differentially expressed genes defined as |fold change| ≥ 2 and adjusted P‑value ≤ 0.05. GO functional enrichment and KEGG pathway analyses were performed on the differentially expressed genes.

### Western blot

RIPA lysis buffer (200 μL) was added per 20 mg of tissue. Lysates were centrifuged at 12 000 × *g* for 5 min, and the supernatant containing total protein was collected. Protein concentration was determined using a BCA assay. Samples were separated by polyacrylamide gel electrophoresis, transferred to a PVDF membrane, and blocked with 5% skim milk for 2 h at room temperature. The membrane was then incubated overnight at 4°C with the CALM1 antibody (1:3000, rabbit, sm‑61141r, Bioss), followed by detection with the corresponding secondary antibody.

### Statistical analysis

The experimental data were statistically analyzed using Origin8.0 software and expressed as the mean ± standard deviation. t-test and ANOVA were used for comparisons between groups. *P < 0.05 indicated a significant difference, and ** *P *< 0.01 indicated a highly significant difference.

## Results and discussion

### Preparation and characterization of PLLA/PEO/Mg_2_Si fibers

**Figure 2:**
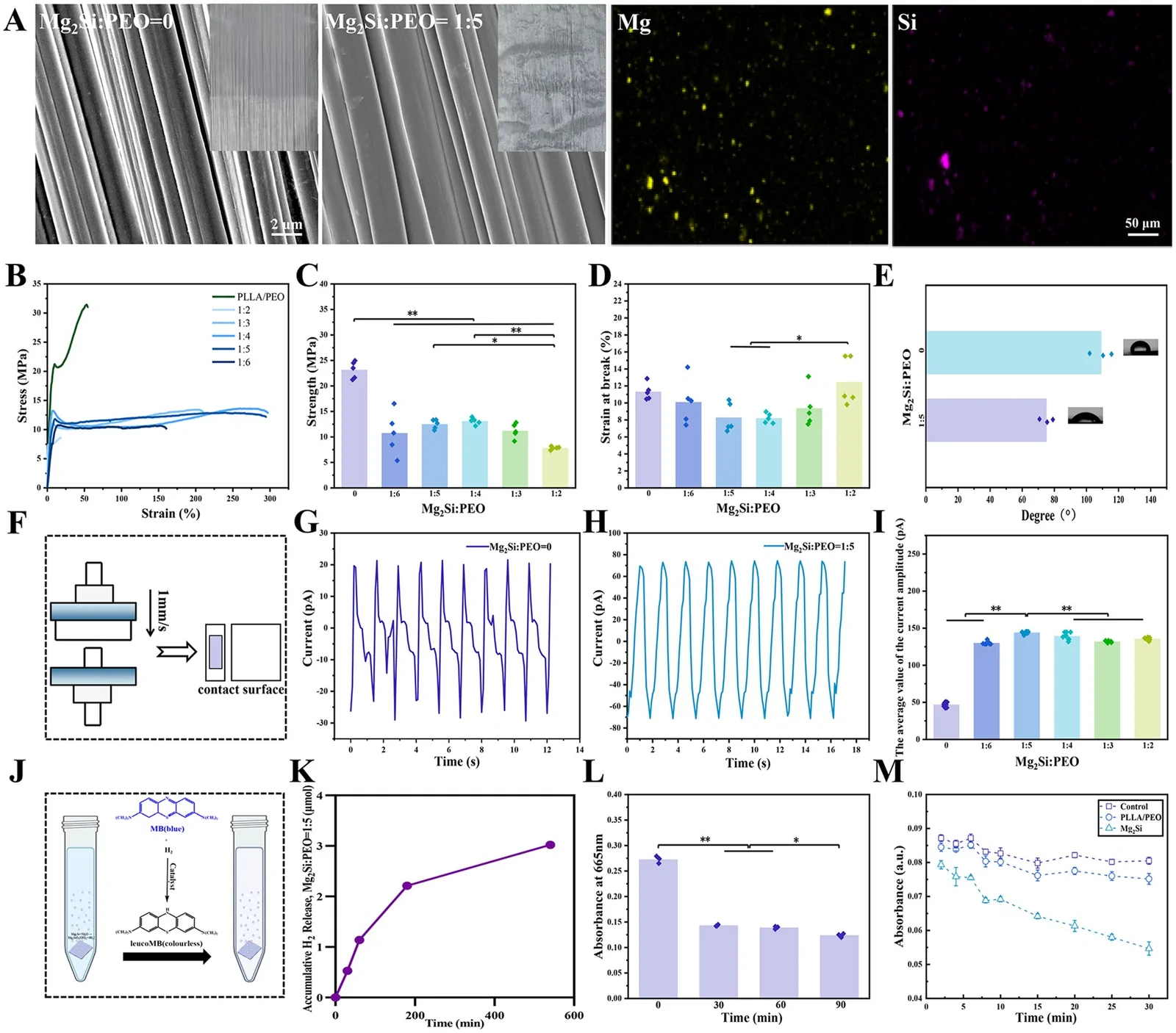
Characterization of electrospun PLLA/PEO/Mg_2_Si fiber membranes. (**A**) SEM image and EDS spectrum, with an inset showing a macrograph for assessing orientation and successful incorporation of Mg_2_Si. (**B**) Tensile stress-strain curves. (**C**) Tensile strength (n = 5). (**D**) Strain at break (n = 5). (**E**) Contact angle (n = 3). (**F**) Schematic of piezoelectric testing. (**G, H**) Piezoelectric output. (**I**) Average current output intensity of the fiber membrane (n = 7). (**J**) Schematic of hydrogen release and MB reduction upon water contact. (**K**) Hydrogen release from the fiber membrane in PBS. (**L**) Hydrogen release and MB reduction in cell culture medium (n = 3). (**M**) Removal of ROS by the fiber membrane in PBS (n = 3). (* *P* < 0.05, ** *P* < 0.01)

This study fabricated linearly aligned piezoelectric nanofiber membranes composed of PLLA, PEO and Mg_2_Si via electrospinning. Scanning electron microscopy (SEM) images (Figure 2A and Supplementary Figure S1A) revealed that both PLLA/PEO and PLLA/PEO/Mg_2_Si fibers exhibited highly unidirectional alignment, with the anisotropic nanotopography providing contact guidance to support biomimetic nerve regeneration [27]. Energy-dispersive X-ray spectroscopy (EDS) elemental mapping (Figure 2A) confirmed successful incorporation of Mg_2_Si, showing distinct silicon and magnesium signals exclusively within PLLA/PEO/Mg_2_Si fibers, while only C and O signals were detected in PLLA/PEO fibers (Supplementary Figure S1A).

Mechanical properties were evaluated to assess scaffold suitability for axonal regeneration [43]. Figure 2B shows stress–strain curves of oriented PLLA/PEO and PLLA/PEO/Mg_2_Si fibers under horizontal tension. PLLA/PEO fibers exhibited significantly higher tensile strength (23.16 ± 1.67 MPa) than Mg_2_Si‑containing fibers. For Mg_2_Si/PEO ratios from 1:6 to 1:2, tensile strengths were 10.75 ± 4.22, 12.49 ± 0.91, 13.09 ± 0.65, 11.19 ± 1.43 and 7.83 ± 0.30 MPa, respectively (Figure 2C). Strain at break remained largely unaffected by Mg_2_Si incorporation (Figure 2D): PLLA/PEO fibers showed 11.32 ± 0.97%, while Mg_2_Si/PEO groups ranged from 8.21% to 12.46%. While Mg_2_Si incorporation compromised the tensile strength relative to pure PLLA, the resulting mechanical properties still surpassed the tensile strength of the native rat sciatic nerve (∼1.4–2.7 MPa), confirming their suitability for peripheral nerve scaffold applications. [44]. Surface wettability was assessed by water contact angle. PLLA/PEO membranes exhibited a vertical contact angle of 109.30 ± 6.86°, indicating hydrophobicity. After Mg_2_Si incorporation, the contact angle significantly decreased to 75.13 ± 4.18° (Figure 2E and Supplementary Figure S1F), reflecting enhanced hydrophilicity, attributed to Mg (OH)_2_ formation as a hydrophilic phase within the fiber matrix [45].

Given the well-documented piezoelectricity of PLLA, we investigated the capacity of the composite fibers to generate electrical signals under mechanical stimulation [28]. Figure 2F–H and Supplementary Figure S1B–1E displays the piezoelectric outputs of fiber membranes with varying compositions. As shown in Figure 2I, PLLA/PEO fibers produced a current amplitude of 46.95 ± 3.10 pA. After Mg_2_Si incorporation, the output current initially increased and then decreased with increasing Mg_2_Si content, peaking at a Mg_2_Si/PEO ratio of 1:5 with an amplitude of 144.11 ± 1.55 pA. This enhancement is likely due to the conductive nature of Mg_2_Si particles, which augment charge transfer at optimal concentrations. However, excessive Mg_2_Si content may disrupt the PLLA matrix and impede piezoelectric charge transmission, establishing an optimal doping range. Furthermore, we validated the voltage output of the PLLA/PEO/Mg_2_Si fibrous membrane under ultrasonic stimulation, which generated stable and reproducible piezoelectric signals (Supplementary Figure S3), confirming its reliable responsiveness to ultrasound exposure *in vitro*. To verify the sensitivity of the composite nanofibers under physiologically relevant low pressures (typically below 1 kPa), we fabricated a 1 cm^2^ piezoelectric sensor using the optimized Mg_2_Si-containing fibers integrated with a polyimide (PI) substrate (Supplementary Figure S4A). The sensor’s output was characterized using an electrochemical workstation. As shown in Supplementary Figure S4C, the sensor responded to mechanical deformation induced by a water droplet (∼20 Pa), generating an output current of approximately 3.26 ± 0.15 pA/cm^2^. Similarly, it detected the mechanical deformation associated with adult respiration (∼700 Pa) and produced an output current of 4.85 ± 2.37 pA/cm^2^ (Supplementary Figure S4E). To further evaluate sensitivity under real physiological conditions, the sensor was applied to various human body sites. Supplementary Figure S4D illustrates its response to blinking-induced deformation, yielding an output current of 1.94 ± 0.28 pA/cm^2^. When attached to the right wrist, the sensor detected subtle arterial pulsations and converted them into distinguishable piezoelectric signals (Supplementary Figure S4B). Collectively, these results demonstrate that the PLLA/PEO/Mg_2_Si piezoelectric nanofibers exhibit exceptional sensitivity to minute mechanical stimuli and can reliably transduce diverse physiological motions into detectable electrical signals [46].

The hydrogen release capacity of PLLA/PEO/Mg_2_Si membranes were evaluated under physiological conditions. As Mg_2_Si reacts with water to generate H_2_, sustained H_2_ release from the composite membrane was measured in PBS (pH 7.4) using a MB/platinum nanoparticle probe system (Figure 2J and K and Supplementary Figure S2A). Intracellular hydrogen release was further examined using the same probe. As shown in Supplementary Figure S2B, a visible reduction in MB blue intensity was observed at 90 min, and the absorbance of MB decreased progressively with prolonged incubation (Figure 2L), confirming sustained hydrogen production and effective reduction of MB [47, 48]. These results validate the scaffold’s capacity for controlled hydrogen release in physiological environments.

POD is a redox enzyme widely found in plants and animals that catalyzes the reduction of hydrogen peroxide (H_2_O_2_) [49]. To evaluate the POD-mimetic activity of Mg_2_Si‑containing fibers, ABTS was used as a chromogenic substrate. As shown in Figure 2M, neither the Control (PBS) nor the PLLA/PEO group exhibited notable absorbance changes. In contrast, the presence of Mg_2_Si led to rapid ABTS oxidation accompanied by a marked decrease in absorbance, indicating that hydrogen generated from Mg_2_Si effectively catalyzes H_2_O_2_ decomposition and exhibits POD‑like activity.

### Fibrous membranes for regulating neuronal behavior

**Figure 3:**
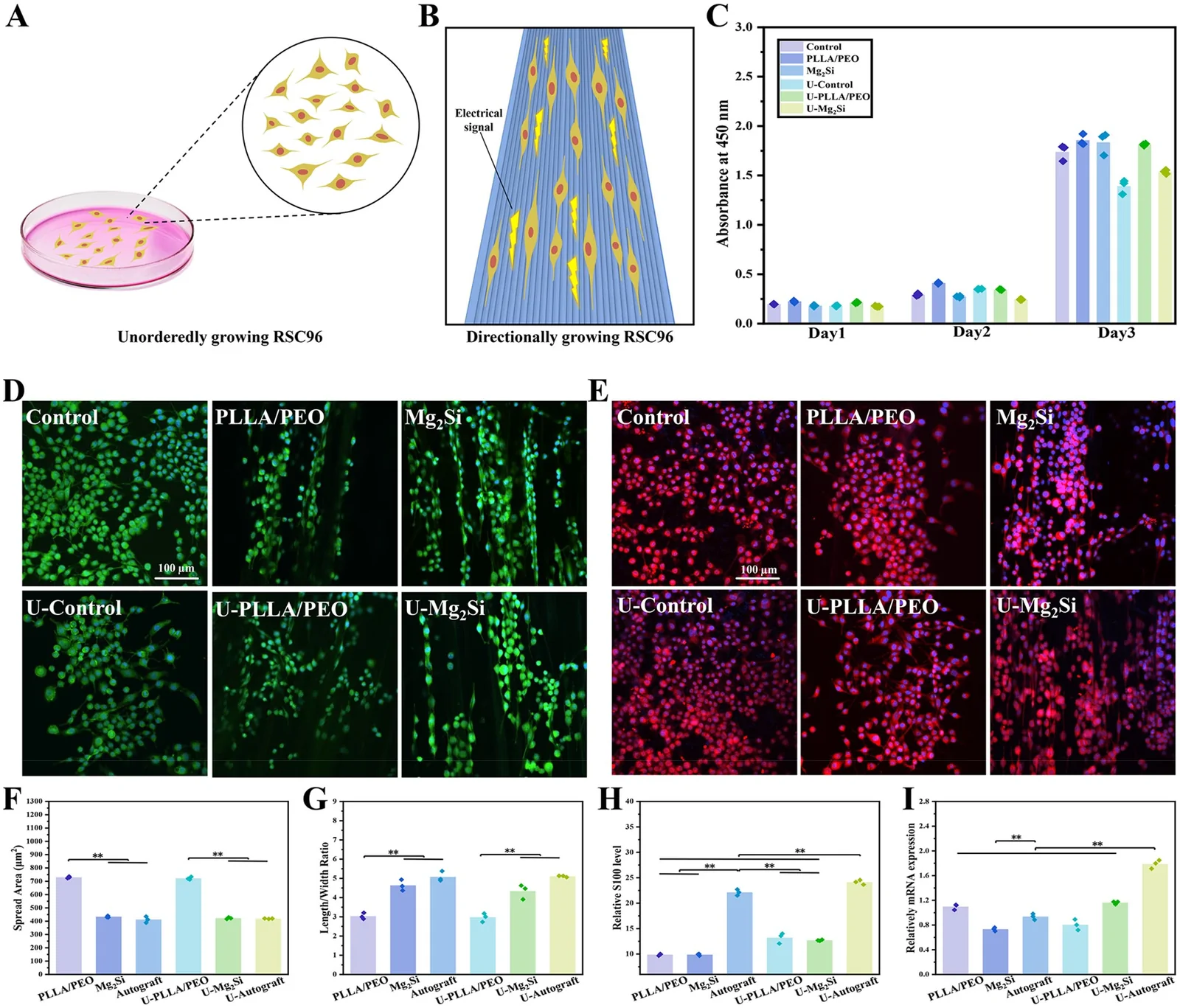
Immunofluorescence staining of RSC96 cells cultured on blank Petri dishes (TCP), PLLA/PEO fiber membranes and PLLA/PEO/Mg_2_Si fiber membranes. (**A**) Schematic of unordered RSC96 growth on a blank Petri dish. (**B**) Schematic of oriented RSC96 growth on fiber membrane. (**C**) CCK-8 cell proliferation assay. (**D**) Cytoskeletal actin staining. (**E**) Immunofluorescence staining for S100 protein. (**F, G**) Spreading area and quantitative analysis of aspect ratios. (**H**) Quantitative analysis of S100 protein fluorescence intensity. (**I**) Quantitative analysis of S100 gene expression. (n = 3, ** *P* < 0.01)

To evaluate the cytotoxicity of PLLA/PEO and PLLA/PEO/Mg_2_Si fibrous scaffolds, RSC96 Schwann cells were co-cultured with each material formulation. Cell proliferation assays (Figure 3C) demonstrated robust and sustained cell growth across all experimental groups, confirming excellent biocompatibility and the absence of cytotoxic effects. Given that piezoelectricity in PLLA-based materials can be effectively induced by ultrasonic stimulation, we applied ultrasound (300 W) to activate piezoelectric output during culture [50]. Pronounced cell proliferation was observed in the U-TCP, U-PLLA/PEO and U-Mg_2_Si groups, indicating that the ultrasound-triggered piezoelectric effect significantly enhances cell growth and proliferation [51]. Cellular morphology on oriented fiber substrates was further characterized. Cells cultured on standard tissue culture plates extended randomly in all directions (Figure 3A), whereas those seeded on aligned fiber membranes exhibited pronounced elongation and orientation along the fiber axis, displaying narrow, spindle-like morphologies (Figure 3B). Cytoskeletal organization was visualized by Actin-Tracker Green-488 staining (Figure 3D). While control groups (Control and U-Control) exhibited typical isotropic spreading, cells on aligned fibers showed reduced spreading area and significantly increased aspect ratios, consistent with contact guidance imposed by the ridge-and-groove nanotopography (Figure 3F and G). Such physical cues are known to direct actin cytoskeleton rearrangement, focal adhesion formation, filopodia extension and directional migration—key processes in Schwann cell alignment and nerve guidance [52, 53]. This oriented architecture facilitates the directed migration of neural cells to the injury site and supports the guided regeneration of damaged nerve fibers.

Following peripheral nerve injury, denervated Schwann cells within Büngner bands upregulate neurotrophic factor secretion and guide regenerating axons toward their targets. To assess functional maturation of Schwann cells on the scaffolds, immunofluorescence staining was performed for S100β, a canonical marker of differentiated Schwann cells (Figure 3E). Consistent with cytoskeletal staining, cells cultured on aligned fibers displayed elongated morphologies oriented along the fiber direction. No significant difference in S100β fluorescence intensity was observed between the Control and U-Control groups, indicating that ultrasound treatment alone does not affect S100β expression (Figure 3H). However, S100β expression was significantly upregulated in the U-PLLA/PEO group compared to the PLLA/PEO group, and similarly elevated in the U-Mg_2_Si group relative to the Mg_2_Si group. Notably, S100β immunoreactivity was highest in the U-Mg_2_Si group, underscoring the synergistic contribution of Mg_2_Si-enhanced piezoelectric signal output to Schwann cell maturation [54]. Real-time quantitative PCR analysis corroborated these findings: after 3 days of culture under ultrasonic stimulation, RSC96 cells seeded on PLLA/PEO/Mg_2_Si fibers exhibited significantly elevated S100β mRNA levels relative to nonstimulated controls (Figure 3I). Together, these data demonstrate that the aligned fiber architecture promotes oriented outgrowth of neurofilaments, while ultrasound-triggered piezoelectric signals actively propel axonal extension and enhance Schwann cell expression of S100β [55–57]. Collectively, these results highlight the synergistic interplay between topographical guidance and piezoelectric modulation in the PLLA/PEO/Mg_2_Si scaffold system. This combined strategy effectively augments Schwann cell function and directs cellular behavior, thereby maximizing the regenerative potential of the scaffold *in vitro*.

### Reactive oxygen species scavenging capacity and induction of macrophage M2 polarization

**Figure 4:**
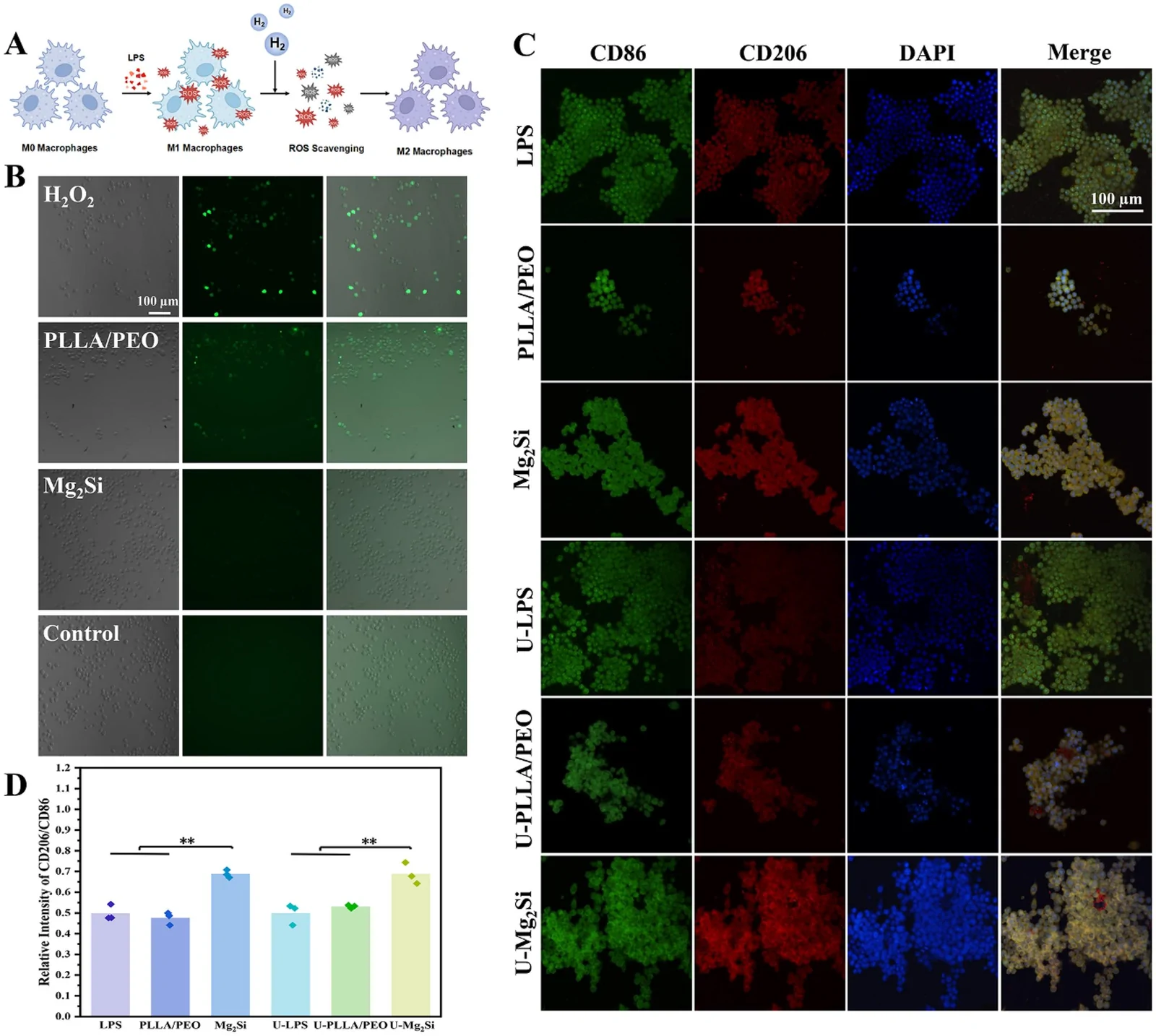
Intracellular ROS scavenging and induction of macrophage M2 polarization. (**A**) Schematic diagram of hydrogen-induced M2 polarization of macrophages. (**B**) Fluorescent images of intracellular ROS staining in macrophages. (**C**) Immunofluorescent staining of macrophages for CD86/CD206/DAPI. (**D**) Statistical graph of CD206/CD86 fluorescence intensity. (n = 3, ** *P* < 0.01)

Although low levels of ROS act as signaling molecules under normal conditions, excessive ROS accumulation triggered by stimuli such as H_2_O_2_ can disrupt cellular homeostasis [58]. Having previously demonstrated that hydrogen generated from Mg_2_Si effectively catalyzes H_2_O_2_ decomposition in PBS, we further assessed the scaffold antioxidant capacity by detecting intracellular ROS levels using the oxidant‑sensitive probe DCFH‑DA. Figure 4A schematically illustrates hydrogen‑mediated macrophage M2 polarization, while Figure 4B shows the ROS‑scavenging efficacy of the PLLA/PEO/Mg_2_Si membrane. Macrophages exposed to H_2_O_2_ exhibited the strongest DCFH‑DA fluorescence, indicating H_2_O_2_‑induced ROS overproduction. Notably, treatment with Mg_2_Si‑containing membranes markedly reduced fluorescence in H_2_O_2_‑stimulated macrophages to levels comparable to untreated controls. These results demonstrate that hydrogen released from Mg_2_Si efficiently neutralizes ROS and other free radicals *in vitro*, highlighting its therapeutic potential for oxidative stress‑associated pathologies [40].

Peripheral nerve injury triggers a complex inflammatory response involving diverse immune cells such as macrophages and neutrophils [59]. In the early phase, immune cells rapidly infiltrate the lesion site to clear debris and contain tissue damage [60]. However, persistent oxidative stress can exacerbate this response through excessive release of cytotoxic mediators, leading to secondary tissue damage, cavitation or fibrosis [61]. Given the central role of macrophages, their phenotypic polarization was assessed by immunostaining for CD86 (M1, pro‑inflammatory) and CD206 (M2, anti‑inflammatory). LPS stimulation markedly increased CD86 expression, confirming successful M1 polarization, with similar results observed in the PLLA/PEO group. In contrast, Mg_2_Si treatment significantly reduced CD86 expression and increased CD206 levels, indicating that hydrogen promotes repolarization toward an anti‑inflammatory phenotype (Figure 4C and D). These effects were consistent regardless of ultrasound application [41]. Together, these findings suggest that H_2_ exerts immunomodulatory effects by modulating macrophage polarization dynamics.

**Figure 5:**
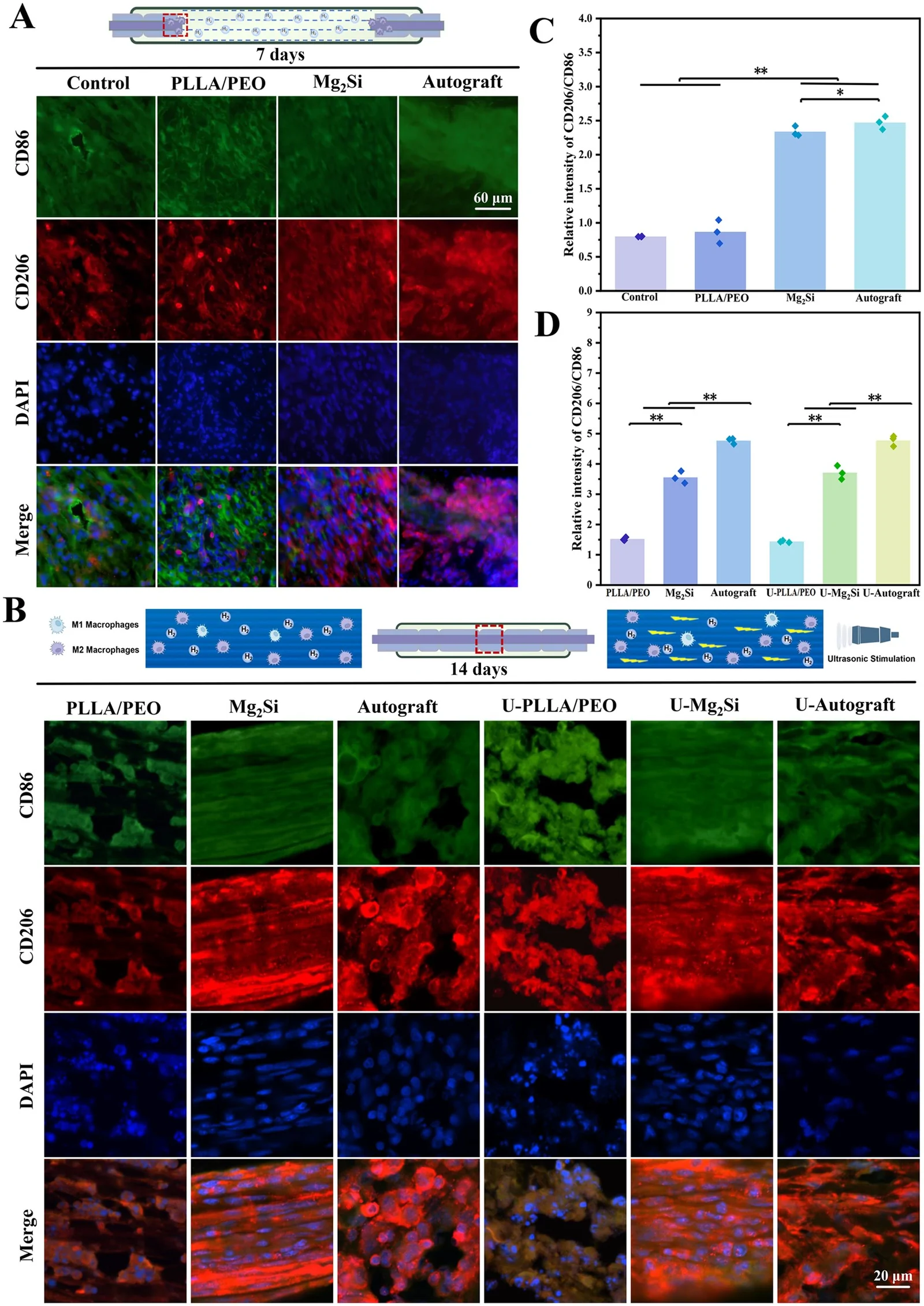
H_2_ induces polarization changes in macrophages *in vivo*. (**A**) Immunofluorescence staining for CD86/CD206/DAPI at the proximal nerve stump 1 week postsurgery. (**B**) Immunofluorescence staining for CD86/CD206/DAPI on regenerating nerves 2 weeks postsurgery. (**C, D**) Histograms of CD206/CD86 fluorescence intensity at 1 and 2 weeks postsurgery. (n = 3, * *P* < 0.05, ** *P* < 0.01)

Inflammation represents the initial stage of the nerve regeneration cascade. Following injury, damage-associated molecular patterns (DAMPs) and potential infections trigger an acute inflammatory response dominated by M1 macrophages, which clear tissue debris and pathogens. As inflammation resolves and immune homeostasis is restored, a phenotypic shift toward M2 predominance occurs, facilitating tissue repair and regeneration [62]. To evaluate the *in vivo* immunomodulatory role of H_2_ in this process, early macrophage polarization was assessed by immunofluorescence staining for CD86 and CD206 at postoperative weeks 1 and 2 (Figure 5A and B). Empty silicone conduits served as negative controls, while autografts served as positive controls. Additional groups included PLLA/PEO and PLLA/PEO/Mg_2_Si conduits. At Day 7, the PLLA/PEO group exhibited lower CD206 expression and was characterized predominantly by M1 macrophages. Quantitative analysis of fluorescence intensity ratios (CD206/CD86) across three randomly selected regions per sample revealed that the proportion of M1 macrophages was significantly higher in the PLLA/PEO group than in the Mg_2_Si group, whereas the proportion of M2 macrophages was significantly lower (Figure 5C). To investigate the potential modulatory effects of ultrasound, rats received ultrasound treatment beginning on Day 7, and regenerated tissue was harvested on Day 14. As the Control group (empty conduits) showed no tissue bridging at 12 weeks postsurgery (Supplementary Figure S5), only the PLLA/PEO, Mg_2_Si, and Autograft groups were evaluated at this time point. The staining results are shown in Figure 5B. By Day 14, macrophage polarization had shifted toward an M2-dominant profile in all groups. Notably, the Mg_2_Si group exhibited significantly higher M2 macrophage proportions and significantly lower M1 proportions compared to the PLLA/PEO group. Ultrasound-treated groups showed consistent trends (Figure 5D). These findings demonstrate that H_2_ released from Mg_2_Si-containing scaffolds effectively promotes *in vivo* macrophage polarization toward the reparative M2 phenotype, thereby attenuating inflammatory responses and accelerating the restoration of immune homeostasis [63].

### PLLA/PEO/Mg_2_Si microfibers for neural regeneration

**Figure 6:**
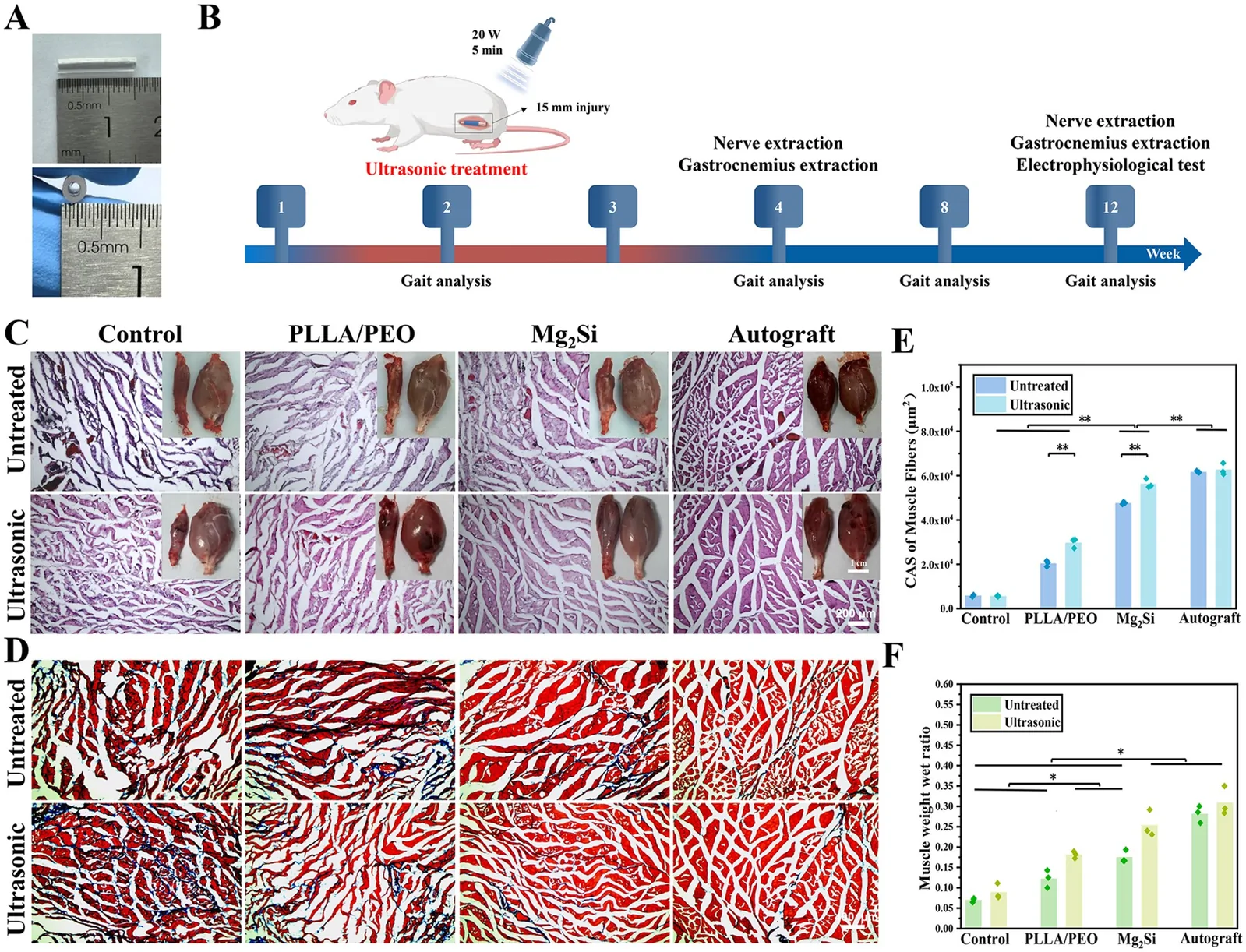
Qualitative and quantitative analysis of the gastrocnemius muscle. (**A**) Fibrous membranes prepared via electrospinning were fabricated into silicone tubes serving as nerve conduits. (**B**) Timeline of animal experiments. (**C**) Physical images of the muscle and its gastrocnemius muscle sections stained with hematoxylin and eosin. (**D**) Physical images of the muscle and its gastrocnemius muscle sections stained with Masson’s trichrome. (**E**) Quantitative analysis of muscle fiber area. (**F**) Wet weight ratio of the muscle. (n = 3, * *P* < 0.05, ** *P* < 0.01)

PLLA/PEO and PLLA/PEO/Mg_2_Si fiber membranes were rolled into tubular constructs and inserted into silicone tubing (outer diameter: 2 mm; inner diameter: 1 mm; length: 15 mm) to provide mechanical support and prevent scaffold collapse during nerve repair (Figure  6A). A rat sciatic nerve transection model with a 15 mm defect was used. The negative control group (Control) received an empty silicone tube, and the Autograft group served as the positive control. Two experimental groups were included: the PLLA/PEO group (PLLA/PEO membrane) and the Mg_2_Si group (PLLA/PEO/Mg_2_Si membrane). Figure  6B outlines the experimental timeline. Rats received ultrasound therapy during weeks 2–3 postsurgery. Paw prints were collected at weeks 2, 4, 8 and 12 for functional recovery analysis. At weeks 4 and 8, gastrocnemius muscles and nerve tissues were harvested to assess repair outcomes.

Following transection, the nerve loses its signal transmission capability, resulting in loss of motor control and subsequent atrophy of the target gastrocnemius muscle [64]. This atrophy is characterized by reduced muscle fiber size and increased collagen deposition, persisting until muscle recovery occurs [65]. Figure 6F presents the wet weight ratio of the gastrocnemius muscle in each experimental group, used as an indicator of muscle atrophy. The left and right sides represent the operated and nonoperated limbs, respectively (Figure 6C). A wet weight ratio approaching 1.00 reflects minimal muscle atrophy. The Injury and PLLA/PEO groups exhibited the lowest wet weights and most pronounced atrophy. The Mg_2_Si group showed a wet weight ratio of 0.176 ± 0.016, significantly lower than that of the Autograft group (0.282 ± 0.021). Notably, the U-Mg_2_Si group achieved a wet weight ratio of 0.254 ± 0.033, comparable to both the Autograft and U-Autograft groups (0.309 ± 0.036). Quantitative analysis of gastrocnemius muscle fibers (Figure 6E) further corroborated these findings: the Control and PLLA/PEO groups displayed loosely arranged muscle fibers with evident intercellular gaps, whereas the Mg_2_Si group exhibited the largest muscle fiber area, second only to the Autograft group. Masson’s trichrome staining (Figure 6D) revealed collagen fibers in blue and muscle fibers in red. In cases of severe atrophy, increased collagen deposition indicated an inflammatory response. The Injury group showed abundant collagen surrounding muscle fibers. In contrast, the U-Mg_2_Si group exhibited effects comparable to the Autograft and U-Autograft groups, with minimal collagen staining, suggesting effective muscle recovery and a mitigated inflammatory response. These findings indicate that the U-Mg_2_Si group effectively promotes nerve bridging and signal transmission, facilitates functional muscle recovery and reduces muscle atrophy.

**Figure 7:**
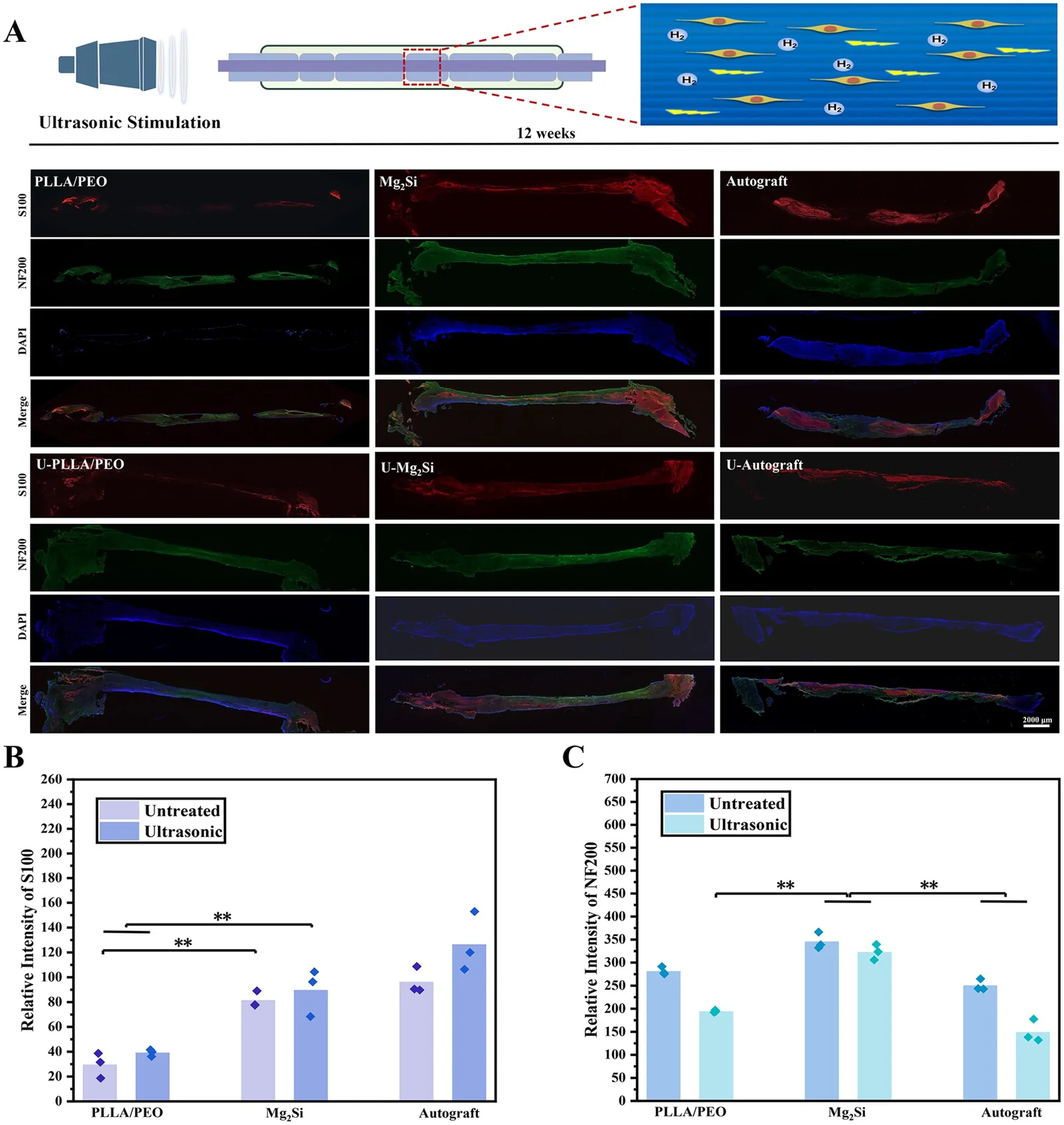
Qualitative and quantitative analysis of NF200 and S100β proteins. (**A**) Immunofluorescence staining of NF200 and S100β proteins in nerve sections from 12-week-old rats’ postsurgery. (**B**) Semi-quantitative analysis of S100β protein expression. (**C**) Semi-quantitative analysis of NF200 protein expression. (n = 3, ** *P* < 0.01)

At 12 weeks post-implantation, histological analysis of regenerated neural tissue was performed to evaluate the scaffold’s role in nerve fiber and axonal regeneration (Figure 7A). Comprehensive nerve regeneration assessment requires consideration of both structural integrity and the extent of axonal and myelin regeneration. Two key markers were employed: S100β (to label Schwann cells) and NF200 (to label neurofilaments in regenerating axons) [66]. As the Control and U-Control groups contained empty catheters with no tissue connectivity (Supplementary Figure S5), only the remaining six groups were subjected to immunofluorescence staining at 12 weeks. S100 protein expression in the U-Mg_2_Si group was significantly elevated compared to the PLLA/PEO group and showed no significant difference from the Autograft and U-Autograft groups (Figure 7B). NF200 fluorescence intensity was 281.477 ± 8.623 in the PLLA/PEO group and 194.396 ± 2.529 in the U-PLLA/PEO group. In the Mg_2_Si group, NF200 expression levels before and after ultrasound treatment were 345.719 ± 18.140 and 322.898 ± 16.843, respectively (Figure 7C), both significantly higher than those in the PLLA/PEO and Autograft groups. These results collectively confirm the beneficial effect of the U-Mg_2_Si group in promoting peripheral nerve injury repair.

### Assessing neural recovery based on rat behavior

**Figure 8:**
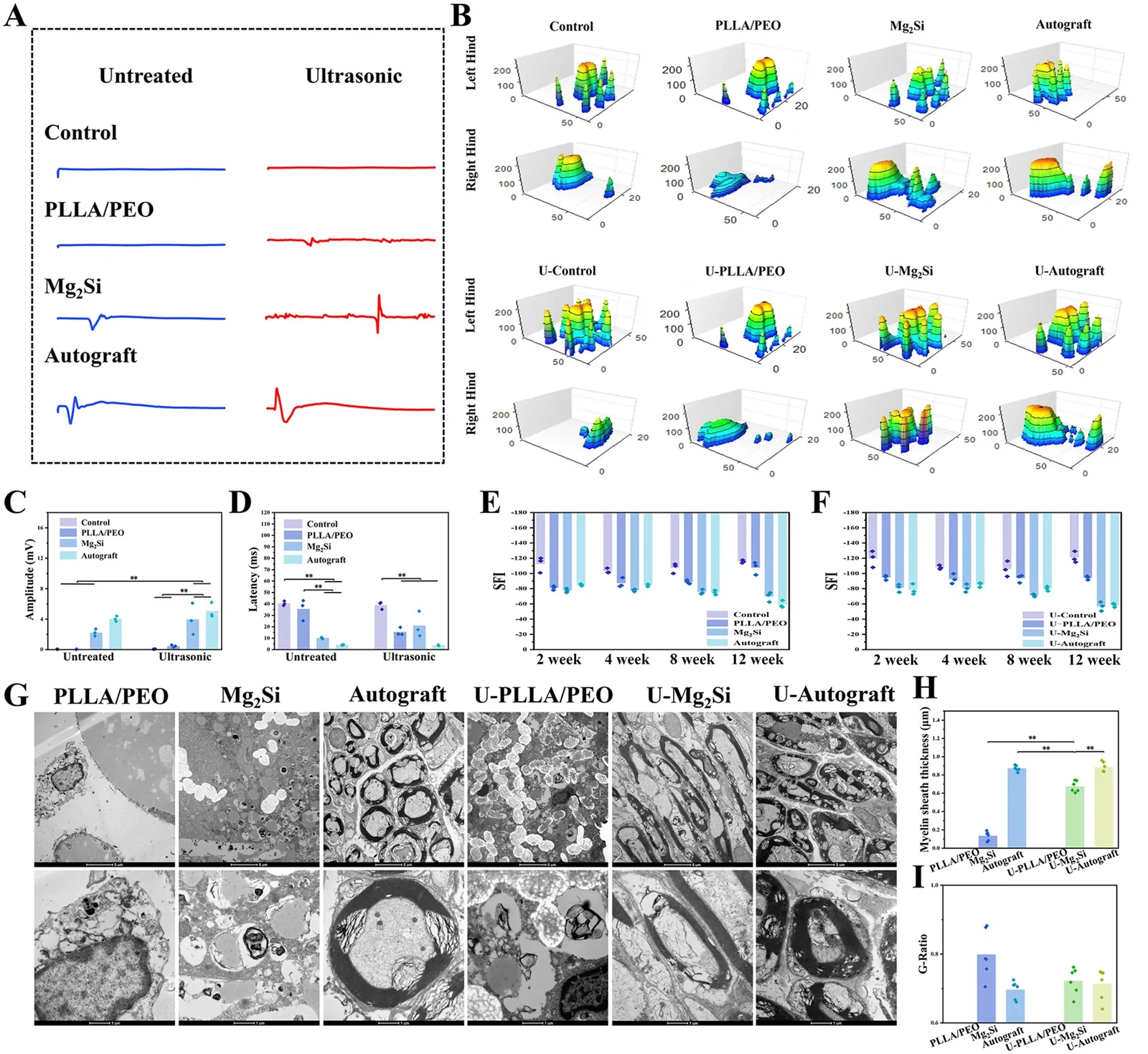
Postoperative 12-week experimental rats. (**A**) Compound muscle action potential (CMAP) recording. (**B**) Footprint test. (**C**) Quantitative analysis of CMAP amplitude (n = 3). (**D**) Quantitative analysis of CMAP latency (n = 3). (**E**) Sciatic nerve function index in rat without ultrasound treatment (n = 3). (**F**) Sciatic nerve function index in rat with ultrasound treatment (n = 3). (**G**) Transmission electron microscopy image of a transverse section of regenerated nerve in rat at 12 weeks postsurgery. (**H**) Quantitative statistics of myelin thickness (n = 6). (**I**) Quantitative statistics of myelin *G* ratio (n = 6). (** *P* < 0.01)

Figure 8B shows footprints recorded using the CatWalk gait analyzer as rats traversed the runway 12 weeks after injury. This device accurately displays footprint contours and force distribution. Figure 8E and F presents the sciatic nerve function index (SFI) values across experimental groups, where –100 indicates complete neurological dysfunction and 0 represents normal sciatic nerve function [65]. In all groups, absolute SFI values progressively declined over time. The U‑Mg_2_Si group exhibited the most favorable nerve function recovery, with an SFI of –69.654 ± 6.360, slightly below that of the Autograft group (–59.265 ± 4.727). Under ultrasound stimulation, the PLLA/PEO/Mg_2_Si group demonstrated the strongest functional recovery, achieving an SFI of –56.789 ± 5.963, comparable to the Autograft group (–58.312 ± 2.454). These results indicate that the PLLA/PEO/Mg_2_Si fibrous membrane neural scaffold conduit effectively improves gait function in rats. Compound muscle action potentials were recorded 12 weeks post‑implantation (Figure 8A). As shown in Figure 8C, the Control and PLLA/PEO groups exhibited markedly lower amplitudes, indicating impaired neural transmission. In contrast, the U‑Mg_2_Si, Autograft and U‑Autograft groups displayed similarly high amplitudes, significantly exceeding those of the other groups. The Control and U‑Control groups showed substantially prolonged nerve latencies relative to all other groups (Figure 8D). Collectively, these findings demonstrate that multifunctional piezoelectric scaffolds effectively promote neural electrical signal conduction and functional recovery.

Given the critical role of myelinated nerve fibers in impulse conduction and sensory function, transmission electron microscopy was employed to assess the regeneration and spatial organization of myelinated fibers in longitudinal nerve sections [43]. Figure 8G illustrates the density of myelinated axons across experimental groups. At 12 weeks, no myelin sheaths were detectable in the PLLA/PEO or U‑PLLA/PEO groups, whereas abundant, well‑defined myelin sheaths were evident in the U‑Mg_2_Si, Autograft and U‑Autograft groups. Myelin thickness, quantified using ImageJ software, measured 0.872 ± 0.031, 0.674 ± 0.060 and 0.885 ± 0.055 μm, respectively. Ultrasonic treatment significantly enhanced myelin thickness in the Mg_2_Si group, indicating that ultrasound‑activated Mg_2_Si scaffolds promote axonal myelination, albeit to a degree still modest relative to the Autograft and U‑Autograft groups (Figure 8H). No significant intergroup difference was observed in the myelin G‑ratio between the Mg_2_Si and Autograft groups, further supporting the efficacy of multifunctional piezoelectric scaffold conduits in facilitating nerve regeneration and myelin formation (Figure 8I).

### Potential mechanisms underlying Mg_2_Si scaffold-mediated neural regeneration

**Figure 9:**
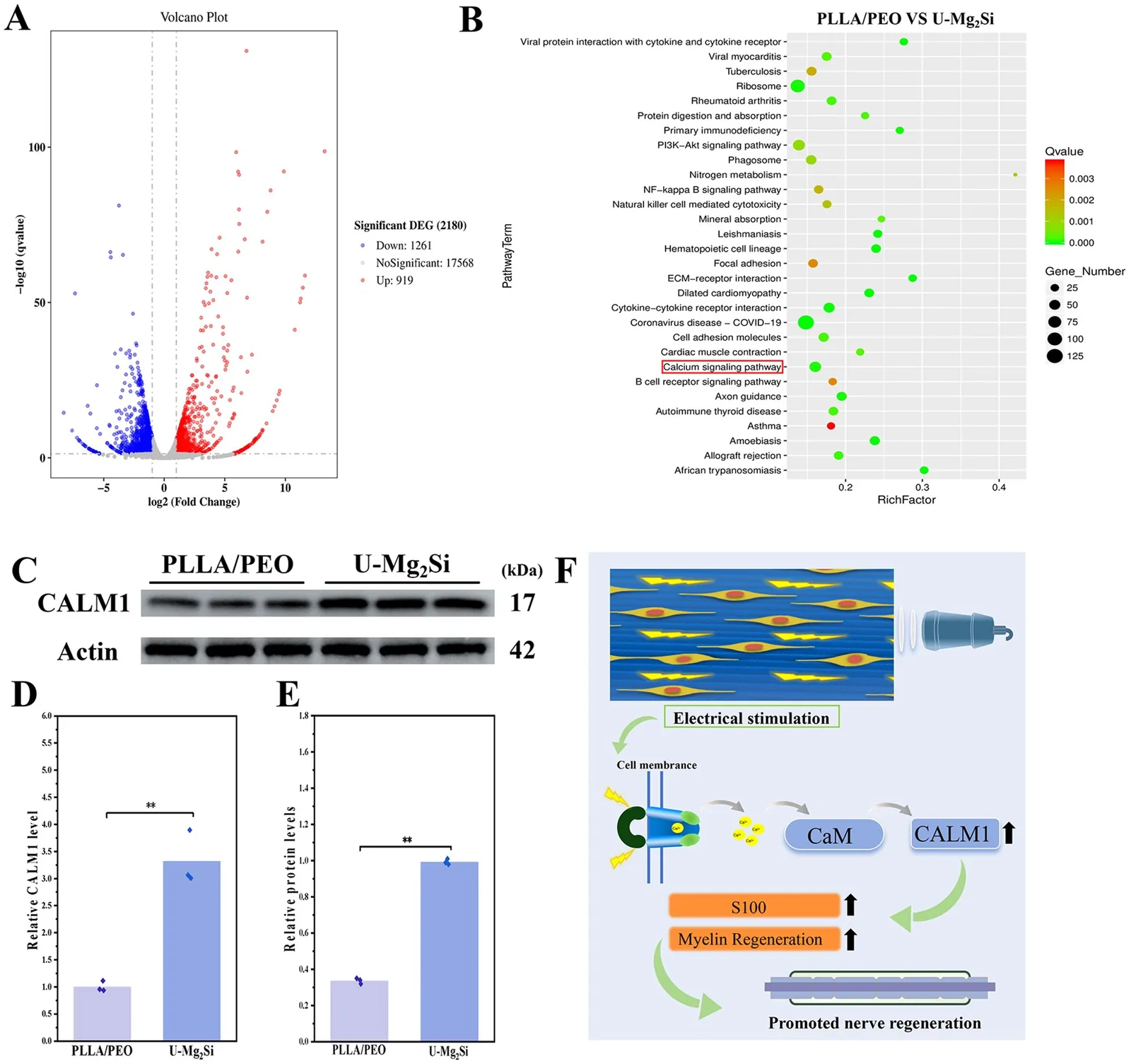
Potential mechanisms promoting peripheral nerve regeneration. (**A**) Volcano plot showing gene expression differences between the PLLA/PEO group and the U-Mg_2_Si group. (**B**) KEGG pathway analysis for the PLLA/PEO and U-Mg_2_Si groups. (**C**) Western blot analysis of neural tissue from the PLLA/PEO and U-Mg_2_Si groups. (**D**) Quantitative analysis of CALM1 gene expression in neural tissue from the PLLA/PEO and U-Mg_2_Si groups. (**E**) Quantitative analysis of Western blot results. (**F**) Schematic diagram of the potential mechanism of piezoelectricity promoting sciatic nerve regeneration. (n = 3, ** *P* < 0.01)

To further investigate the potential mechanisms by which these materials promote neuroregeneration, RNA sequencing analysis was performed on regenerated neural tissue at 12 weeks. Volcano plots revealed differences between the PLLA/PEO group and the U-Mg_2_Si group (Figure 9A). Comparative analysis of RNA sequencing data from the PLLA/PEO and U-Mg_2_Si groups identified various pathways related to nerve regeneration (Figure 9B), with significant enrichment of the calcium signaling pathway. This observation aligns well with the GSEA analysis, which demonstrated positive regulation of the calcium signaling pathway (Supplementary Figure S6). Notably, CALM1, a leading-edge gene in the GSEA results, exhibited significant upregulation. These findings suggest a potential involvement of the calcium signaling pathway in the regenerative process following nerve injury. qPCR results confirmed (Figure 9D) that the U-Mg_2_Si group promoted CALM1 expression. Western blot analysis similarly validated (Figure 9C and E) the higher expression of CALM1 protein. CALM1 encodes calmodulin (CaM), a key intracellular Ca^2+^ sensor that translates Ca^2+^ concentration changes into downstream signaling cascades [67, 68]. During peripheral nerve regeneration, activation of calcium signaling is closely associated with Schwann cell proliferation, migration, myelination and axonal outgrowth [69]. Consistent with these findings, the U-Mg_2_Si group exhibited enhanced S100 expression, thicker myelin sheaths observed by TEM, and improved electrophysiological recovery. Collectively, the transcriptomic, qPCR, and protein expression analyses indicate that activation of calcium signaling may be associated with the enhanced regenerative outcomes observed in the U-Mg_2_Si group. Given the multifunctional nature of the scaffold system, including scaffold alignment, Mg_2_Si incorporation, ultrasound stimulation and potential piezoelectric responses, the present findings suggest a possible involvement of calcium signaling and CALM1 upregulation in mediating the observed biological effects. A schematic working model summarizing the proposed mechanisms is presented in Figure 9F.

Despite the promising results, several limitations should be acknowledged, which in turn inform our future directions. First, the undefined individual contributions of topography, piezoelectricity and H_2_ release call for systematic mechanistic studies with additional control groups. Second, the short-term *in vitro* H_2_ release and uncertain long‑term stability of Mg_2_Si motivate further optimization of scaffold composition and degradation kinetics. Third, the practical challenges of the ultrasound protocol in clinical settings highlight the need for more patient‑friendly stimulation strategies. Fourth, the limited resolution of immunofluorescence images underscores the importance of employing advanced imaging techniques. Fifth, the exclusive use of a rat model necessitates validation in larger animals to establish translational feasibility. Sixth, the lack of direct *in vivo* piezoelectric measurement encourages the development of novel *in situ* detection methods. Beyond these refinements, given its ability to modulate neuronal activity and oxidative stress, this scaffold strategy may also hold promise for other neurological disorders, such as spinal cord injury, traumatic brain injury and neurodegenerative diseases, including Parkinson’s and Alzheimer’s.

## Conclusions

Addressing the critical clinical challenges of difficult-to-reconstruct regenerative microenvironments and slow nerve regeneration following long-distance peripheral nerve injury, this study successfully fabricated a PLLA/PEO/Mg_2_Si biomimetic nerve scaffold using electrospinning technology. An integrated therapeutic system based on ‘piezoelectric–topographic–hydrogen’ synergistic effects was established to promote peripheral nerve regeneration. SEM confirmed the scaffold’s well-aligned fibrous architecture. The incorporation of Mg_2_Si significantly enhanced its piezoelectric output. Leveraging the inherent electrical activity of neural tissue and the essential role of bioelectric signals in nerve repair, these properties collectively establish a tailored physical microenvironment conducive to neural regeneration. *In vitro* cellular assays demonstrated the scaffold’s excellent biocompatibility, its capacity to direct oriented cell adhesion and growth, and its significant upregulation of S100 protein—a specific marker of Schwann cells—thereby providing a cellular foundation for nerve regeneration. Further characterization revealed that the scaffold enables sustained, low-level hydrogen release in cell culture systems while efficiently scavenging ROS. *In vitro*, it promoted macrophage polarization toward the anti-inflammatory M2 phenotype. *In vivo*, it effectively suppressed early inflammatory responses following rat sciatic nerve injury, establishing a favorable immune microenvironment for subsequent nerve regeneration. In a rat model of 15 mm long-distance sciatic nerve transection injury, the scaffold exhibited outstanding reparative efficacy, promoting axonal regeneration and mature myelin formation, mitigating gastrocnemius muscle atrophy, restoring sciatic nerve function and electrophysiological conduction properties, and enhancing both structural and functional recovery of the injured nerve. To further elucidate the underlying molecular mechanisms, transcriptomic analysis was performed on regenerated nerve tissues at 12 weeks post-injury. The piezoelectric material group exhibited significantly elevated expression of CALM1, which encodes CaM—a central regulator of Ca^2+^ signaling. This transcriptional upregulation was corroborated at both the mRNA (qPCR) and protein (Western blot) levels. These findings suggest that the PLLA/PEO/Mg_2_Si scaffold may promote nerve repair, at least in part, through activation of calcium signaling pathways and upregulation of CALM1 expression. In summary, the PLLA/PEO/Mg_2_Si bioinspired neural scaffold developed in this study integrates the synergistic advantages of piezoelectric stimulation, topographical guidance and hydrogen-mediated antioxidant and anti-inflammatory effects to address multiple key challenges in long-distance peripheral nerve repair. This work provides novel mechanistic insights and robust experimental evidence to support clinical translation in peripheral nerve injury treatment and may also inform research into the pathogenesis of related neurodegenerative disorders, underscoring its significant potential for translational application.

## Supplementary Material

rbag156_Supplementary_Data

## Data Availability

The data that support the findings of this study are available from the corresponding author upon reasonable request.
