# Supplementary material for: Piezoelectric-hydrogen synergy in electrospun oriented fibrous scaffold enables multimodal repair of long-distance peripheral nerve defect
Source: Regen Biomater. 2026 Jul 21;13:rbag156. doi: 10.1093/rb/rbag156 (PMC13541378; doi:10.1093/rb/rbag156)
Supplement: Supplementary file 1 [file rbag156_supplementary_data.zip › supplement.docx]

Supporting Information

**Figure S1** (**A**) SEM and macroscopic images of PLLA/PEO/Mg₂Si fibers at different ratios, along with EDS spectra of PLLA/PEO/Mg₂Si fibers. (B-E) Piezoelectric output.

(F) Contact angle.

**Figure S2** (**A**) Hydrogen release profiles of PLLA/PEO/Mg₂Si fibers at different ratios. (**B**) Observed intracellular H₂ release (blue reduction phenomenon) within 90 minutes of incubation with RSC96 cells.

**Figure S3** Piezoelectric output signals generated by fibers under ultrasound stimulation.

**Figure S4** (**A**) Sensor fabricated using a PLLA/PEO/Mg₂Si fiber membrane combined with PI. (**B**) Actual image of the sensor attached to the right wrist and the resulting piezoelectric signal. (**C**) Actual image of the water droplet experiment and the resulting piezoelectric signal. (**D**) Sensor attached to the human eyelid region and the piezoelectric signal generated during blinking. (**E**) Actual image of the breath experiment and the resulting piezoelectric signal.

**Figure S5** Physical image of neural tissue samples collected at 12 weeks.

**Figure S6** GSEA analysis plot of the U-Mg₂Si group.
